# Cortical Activity during a Highly-Trained Resistance Exercise Movement Emphasizing Force, Power or Volume

**DOI:** 10.3390/brainsci2040649

**Published:** 2012-11-20

**Authors:** Shawn D. Flanagan, Courtenay Dunn-Lewis, Brett A. Comstock, Carl M. Maresh, Jeff S. Volek, Craig R. Denegar, William J. Kraemer

**Affiliations:** 1 Human Performance Laboratory, Department of Kinesiology, University of Connecticut, Storrs, CT 06269, USA; Email: shawndflanagan@gmail.com (S.D.F.); courtenay.lewis@uconn.edu (C.D.-L.); brett.a.comstock@gmail.com (B.A.C.); carl.maresh@uconn.edu (C.M.M.); jeff.volek@uconn.edu (J.S.V.); craig.denegar@uconn.edu (C.R.D.); 2 Department of Physiology and Neurobiology, University of Connecticut, Storrs, CT 06269, USA

**Keywords:** exercise, brain, neuroplasticity, motor control, physical activity, movement related cortical activity, electroencephalogram (EEG), fatigue, force, power

## Abstract

Cortical activity is thought to reflect the biomechanical properties of movement (e.g., force or velocity of movement), but fatigue and movement familiarity are important factors that require additional consideration in electrophysiological research. The purpose of this within-group quantitative electroencephalogram (EEG) investigation was to examine changes in cortical activity amplitude and location during four resistance exercise movement protocols emphasizing rate (PWR), magnitude (FOR), or volume (VOL) of force production, while accounting for movement familiarity and fatigue. EEG signals were recorded during each complete repetition and were then grouped by functional region, processed to eliminate artifacts, and averaged to compare overall differences in the magnitude and location of cortical activity between protocols over the course of six sets. Biomechanical, biochemical, and exertional data were collected to contextualize electrophysiological data. The most fatiguing protocols were accompanied by the greatest increases in cortical activity. Furthermore, despite non-incremental loading and lower force levels, VOL displayed the largest increases in cortical activity over time and greatest motor and sensory activity overall. Our findings suggest that cortical activity is strongly related to aspects of fatigue during a high intensity resistance exercise movement.

## 1. Introduction

Exercise is increasingly recognized for its role in optimizing cognitive function and learning, as well as in the prevention and management of neurodegenerative disorders [1,2]. Likewise, it is well established that the brain is essential for neuromuscular adaptations to exercise [3]. While we have begun to appreciate the relationship between the brain and exercise, investigations of brain activity during intense, whole-body exercise are rare. Most brain imaging devices cannot capture brain activity *during* high-intensity, whole-body movements due to blood flow changes or limitations in permitted movement during data acquisition [4]. As a result, many investigations to date have examined brain activity *before* or *after* exercise. In addition, most studies have used isolated (e.g., finger flexion), isometric, or low-intensity movements not typically found in traditional exercise or the behavioral repertoire. 

While investigations of movement may not relate entirely to vigorous exercise, they indicate a cortical representation of the biomechanical properties of movement. Examples of these properties include force (e.g., lifting a heavy box), power (e.g., jumping), or repetition volume (e.g., lifting a box many times). From the standpoint of muscle recruitment, high power and force movements require higher threshold, fast-twitch (or Type II) motor units (an alpha motor neuron and the muscle fibers it innervates) while higher volume movements utilize lower threshold, slow-twitch (or Type I) motor units [5]. As force and power are generated through the orderly recruitment of higher threshold motor units (this concept is known as the *size principle* [6]), higher force and power require the recruitment of larger amounts of muscle mass. In power movements, a quickly accelerated mass is moved at a high velocity (e.g., shot put, sprinting, vertical jump). During this type of ballistic exercise, motor units are recruited earlier and force is produced using three times the number of motor units involved in non-ballistic movements using the same mass [7,8]. 

Since higher force and power have been accompanied by higher cortical (EEG) and elbow flexor (electromyography (EMG)) activity *during* the execution of an elbow flexion task, others have concluded that cortical activity represents the controlling signal of movement [9]. Supporting this theory, elevated cortical activity has been observed with higher force and power in investigations of plantar flexion [10], finger, and hand movements [11,12]. Increases in activity are typically observed above areas thought to play a key role in the control and execution of movement, including the supplementary motor area, primary motor cortex, and sensorimotor cortex [13,14,15,16,17,18,19]. Cortical activity thus appears to reflect the size principle, as it has coincided with the recruitment of the higher threshold motor units (and thus greater muscle mass) responsible for higher power and force production.

In addition to the differences between the biomechanical properties of movement, a fundamental and often independently studied component of exercise research is the impact of fatigue. Most investigations have found progressive increases and widespread changes in cortical activity during repetitive movements [4,15,20,21,22,23,24,25,26]. However, these investigations have varied in their use of incremental or non-incremental loading, the biomechanical aspects of movement, and whether or not fatigue was a specific outcome variable. Brummer *et al.* [4], recently demonstrated that EEG can effectively examine intense whole-body exercise through the use of newer artifact reduction and processing technologies. In his investigation, cortical activity density in specific locations was measured during a dynamic, whole-body cycling exercise movement. In agreement with previous investigations, activity above the primary motor cortex increased with incrementally higher intensity. However, the use of incremental loading in that investigation makes it difficult to determine whether changes in cortical activity can be primarily attributed to increased intensity. Taken together, these investigations suggest that fatigue plays a role in motor and sensory cortical activity, although the extent of this influence is uncertain.

Another increasingly well-recognized aspect of motor activity is the participants’ experience with a given movement. The motor cortex plays a fundamental role in the acquisition and development of new motor skills and this is reflected in cortical activity [16,27,28,29,30]. This theory was highlighted in recent work which showed that improvements made during training could not be retained when transcranial magnetic stimulation (TMS) was applied to the motor cortices of subjects attempting to develop increased muscular force in a finger movement [16]. The central role of learning in the motor cortex is important because the movements used in many investigations were unknown to subjects; cortical activity may have therefore partially reflected training status, rather than the particular movement features of interest. In support of this concept, Karni [30] demonstrated with functional magnetic resonance imaging (fMRI) that subjects who practiced a standard finger movement pattern experienced acute and prolonged growth in the intensity and area of motor cortical activity over three weeks of training. Using EEG in a single-leg press exercise, Falvo *et al.* [28] found that training resulted in more rapid peaks and overall decreases in the magnitude of cortical activity. In another investigation, highly-trained athletes showed decreases in primary motor activity during an exhausting isometric handgrip exercise when compared to non-athletes, who exhibited increased activity at exhaustion [31]. Cortical activity is therefore affected by movement novelty and training, and this may be a function of motor unit activation efficiency, precision, and the biomechanical properties of movement. Thus, the use of untrained subjects may confound other independent variables if left unaddressed.

In summary, the available research suggests that biomechanical properties of movement are responsible for changes in cortical activity before, during, and after exercise, although further examination of cortical activity during whole-body exercise is needed. While fatigue may generally result in increased cortical activity during exercise, how force, power, and volume of movement relate to cortical activity during exercise is unclear. Furthermore, an examination of these factors in an investigation that accounts for subjects’ training status is required to address the potential influence of movement experience. The purpose of the present investigation was to examine changes in cortical activity in primary motor and sensory regions during four resistance exercise movement protocols that emphasized rate (PWR), magnitude (FOR), or volume (VOL) of force production, while accounting for movement familiarity and fatigue. We hypothesized that: (1) if cortical activity is a central representation of the size principle, the highest levels of motor and sensory activity should accompany FOR and PWR protocols; and (2) if movement related cortical activity increases with fatigue, the largest increases in motor activity should occur during the VOL protocol.

## 2. Results

As a general note, the terms *motor* and *sensory* apply to activity in premotor/primary motor and sensory/parietal regions, respectively, as primary motor activity was not statistically different from premotor activity over time, nor was parietal activity different from sensory activity. Additionally, although EEG monitors the ion currents associated with neural activity, both inhibitory and excitatory potentials may be detected as negative or positive depending on where they are generated [32,33]. For each subject and protocol, polarity was typically established from the first repetition and few subjects crossed from negative to positive. Indeed, one subject exhibited predominantly negative activity even during the CTRL protocol. Since the source of signal polarity cannot be determined with a reasonable degree of certainty and because rectified data better depicted the trends in the data, rectified data are presented [34].

### 2.1. Physiological Measures of Exertion

Ratings of perceived exertion (RPE) were lowest in CTRL (0.1 ± 0.2 throughout all sets) and increased in PWR (3.3 ± 1.7 after the first set and 5.9 ± 1.7 after the last set). The highest ratings were seen in FOR (6.3 ± 2.2 after the first set and 9.1 ± 2.5 after the last set) and VOL (6.1 ± 2.3 after the first set and 9.3 ± 2.1 after the last set). No significant differences were seen between FOR and VOL protocols, but all other pairwise differences were significant. Heart rate, another measure of exertion, reached 169.5 ± 3.2 BPM during the VOL protocol, which was significantly higher than PWR (139.9 ± 2.6 BPM) or CTRL (76.0 ± 0.9 BPM) but not FOR (157.1 ± 3.2 BPM). As shown in Table 1, lactate levels following the VOL protocol (IP) rose as high as 21.5 mmol/L. All pairwise differences between protocols were significant immediately post exercise, from PWR to FOR to VOL. Complete procedures and results for measures of tissue disruption (myoglobin, creatine kinase) and physiological stress (cortisol) are reported elsewhere [35]. As described in our previous work, all biochemical measures were highest in the VOL protocol. Thus VOL elicited the greatest physiological and perceptual response, followed by FOR, PWR, and CTRL. 

### 2.2. Sensory and Motor Activity and Biomechanics

**Figure 1:**
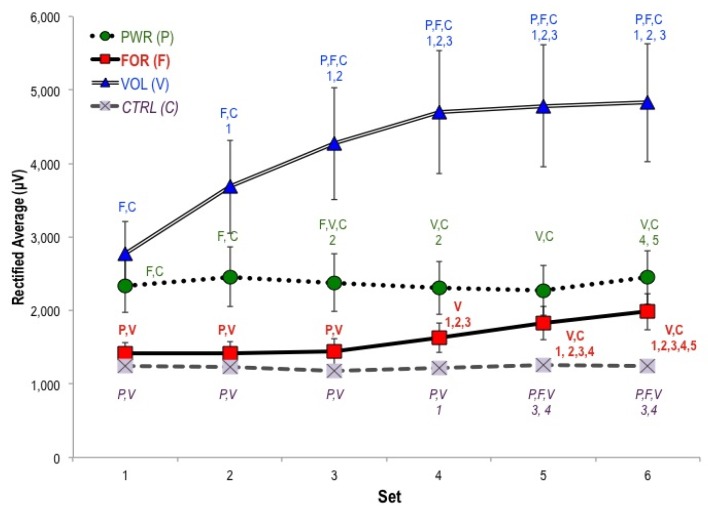
Changes in primary motor activity (C3, CZ, C4) across six sets. By Set 4, the high volume protocol (VOL, ten repetitions per set at 80%) was significantly higher than all other protocols and its first three sets. Please note that the high force protocol (FOR, three repetitions per set at 95% 1RM) begins as a low negative value and is significantly different from all protocols at Set 1. By Set 6, FOR is significantly different from its first four sets and only significantly different from the VOL protocol. Pairwise differences are indicated in the form *X:Y*, indicating *Reference Protocol: Significantly Different Protocol*. “F” indicates the FOR protocol, “V” VOL, and so forth. In a given protocol, “1” indicates the value is significantly different from Set 1 of that protocol, “2” from Set 2, and so forth.

**Figure 2:**
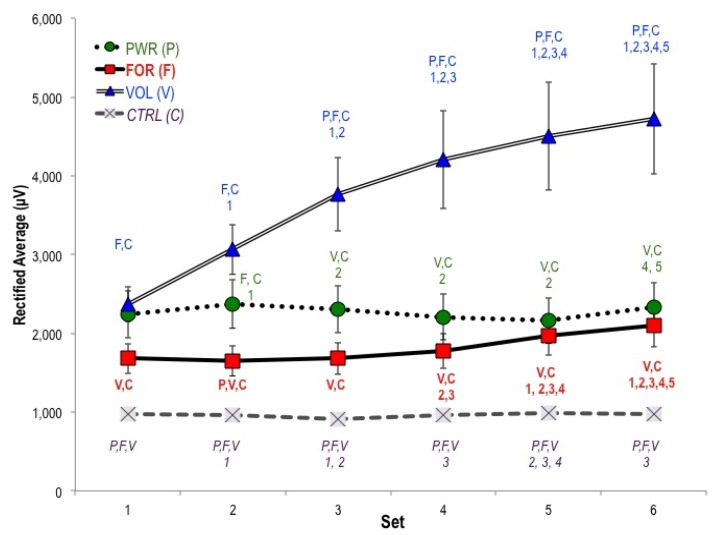
Changes in sensory activity (CP3, CPZ, CP4) across six sets. Sensory activity was especially pronounced in the PWR protocol (30% 1RM jump squats) initially but was rapidly overwhelmed by VOL. Pairwise differences are indicated in the form *X:Y*, indicating *Reference Protocol: Significantly Different Protocol*. “F” indicates the FOR protocol, “V” VOL, and so forth. In a given protocol, “1” indicates the value is significantly different from Set 1 of that protocol, “2” from Set 2, and so forth.

We expected to observe the greatest activity above motor regions in the force (FOR) and power (PWR) protocols (please see Table 2), since these protocols produced the most muscular force and power. Instead, average activity above motor and sensory regions was highest in the VOL protocol across all sets (significantly higher than FOR and CTRL from set one) (Figure 1 and Figure 2). By contrast, activity above the motor region in FOR was not significantly different from CTRL until set five—but its sensory activity did differ from CTRL throughout. Motor and sensory activities were highest in VOL, followed by PWR. Motor and sensory activities were low and relatively constant over time in the CTRL protocol as expected given the lack of stimulus.

**Table 1:** Lactate responses (mmol L^−1^) to protocols prior to exercise (PRE), immediately post (IP), at 30 min post (+30), and at 24 h post (+24). Please note lactate levels immediately post exercise (IP), where pairwise differences exist between all protocols with the exception of CTRL and PWR. The VOL protocol is a source of particular physiological stress (IP 15.95 ± 3.75 mmol L^−1^). ^#^ significant difference from resting and * significant difference from *all* other protocols at 0.05 level.

Protocol	PRE	IP	+30	+24
**PWR**	1.49 ± 0.46	3.01 ± 1.54	1.60 ± 0.51	1.27 ± 0.48
**FOR**	1.37 ± 0.16	8.66 ± 2.81 ^#^ *	2.97 ± 1.03	1.43 ± 0.49
**VOL**	1.29 ± 0.24	15.95 ± 3.75 ^#^ *	8.92 ± 2.98 ^#^ *	1.27 ± 0.41
**CTRL**	1.40 ± 0.40	1.20 ± 0.21	1.08 ± 0.23	1.30 ± 0.29

**Table 2:** Average physical characteristics of each protocol. Protocols were designed to produce maximal values for the biomechanical property of interest. * Significantly greater than *all* other protocols. (The CTRL protocol was not included in the analysis).

Measure	PWR	FOR	VOL	CTRL
Force (N)	2541.58 ± 314.17	3018.21 ± 477.19 *	2539.12 ± 279.61	975.38 ± 100.32
Velocity (m/s)	1.76 ± 0.13 *	0.65 ± 0.05	0.65 ± 0.06	0.00
Power (W)	4355.83 ± 665.62 *	1943.86 ± 372.62	1666.56 ± 288.06	0.00
Volume (repetitions)	18	18	60 *	0.00

### 2.3. Fatigue and Protocols

We hypothesized that motor and sensory activity would increase with fatigue. By set three, VOL displayed significantly greater activity in both regions than all other protocols. Although its change was less dramatic, FOR also displayed increasing activity over time above motor and sensory regions. Muscular power and force decreased most significantly in VOL and FOR, which were also characterized by the largest increases in heart rate, perceived exertion, and physiological measures of exertion (for lactate values, see Table 1). By comparison, motor and sensory activity changed little over time in PWR and CTRL. Thus VOL and FOR displayed the most pronounced increases in activity above motor and sensory regions over the course of protocols.

We hypothesized that higher-threshold motor unit activation during FOR and PWR protocols would be accompanied by greater cortical activity. Contrary to our hypothesis and previous work, from the very first set, the highest levels of activity tended to accompany the VOL protocol. In agreement with past investigations, the largest increases in activity over time occurred in the VOL protocol. In addition, the FOR protocol changed over time to a greater extent than PWR and CTRL.

## 3. Discussion

### 3.1. Biomechanical Properties Secondary in Cortical Activity during Highly-Trained Exercise

Previous work has suggested that the size principle is a key peripheral factor in cortical activity during movement [4,9,10,11,12]. These investigations have observed parallel increases in cortical activity and force or power when increased progressively or in separate trials [4,9,10,11,12,36]. We observed distinct levels and patterns of motor activity in protocols emphasizing power, force, or volume of movement, but there are a number of reasons to question whether cortical activity during exercise primarily reflects the level of muscle activation. 

Accumulated fatigue provided the clearest indication to the contrary. In fatiguing VOL and FOR protocols, motor activity increased despite progressive decreases in power output and resistance loads (which would suggest the loss of contribution from fatigue-prone high threshold motor units). Conversely, motor activity was relatively constant (albeit high) in PWR, where power output was maintained and indices of fatigue were minimal, despite the production of maximal mechanical power (which requires the highest threshold motor units). Increased cortical activity seen in the VOL and FOR protocols with decreased performance indicates that fatigue was well represented in cortical activity. 

Fatigue was expected to interact with cortical activity but a surprising finding was that motor activity in VOL was higher on average than FOR and PWR from the very first set—despite significantly lower levels of force and power. As we discuss in section 3.2, this could highlight the importance and specificity of movement familiarity. In addition, cortical neurons may interact with skeletal muscle through other signaling mechanisms, including synchronization and frequency adjustments [37]. Nonetheless, our investigation has demonstrated that even when fatigue is minimal, cortical activity reflects the biomechanical properties of movement and other factors during a highly-trained whole-body resistance exercise movement.

### 3.2. Fatigue Well Represented in Cortical Activity

**Figure 3:**
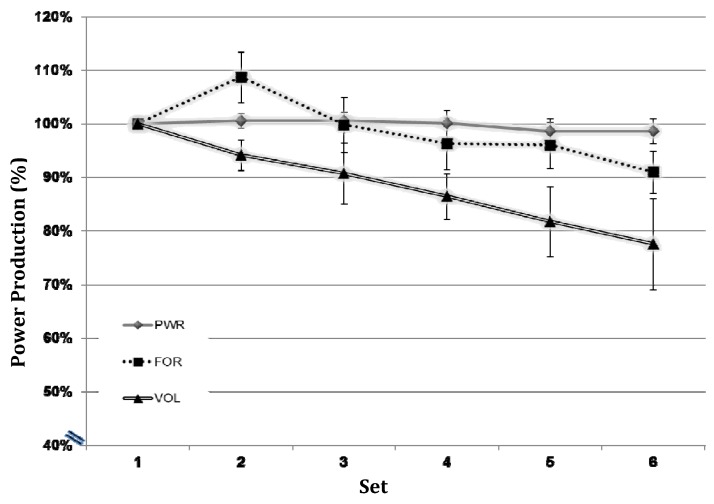
Mean performance over time.

VOL was created to equate the non-independent biomechanical properties of FOR and PWR while incorporating the number of repetitions needed to produce the most load volume and fatigue. VOL and FOR protocol were more fatiguing than PWR and CTRL based on lactate, heart rate, perceived exertion, and performance decrement (See Table 1, Figure 3, and [35]). We hypothesized that if cortical activity increases with fatigue during exercise, the largest increases in activity would occur in VOL, and this was the case. Past investigations have also observed increased motor activity with progressive fatigue while physical output remained constant or decreased. Schillings [26] observed primary motor activity increases before grip contractions performed for 30 min at 70% MVC; force was reduced to 93% of initial levels at the end of the protocol. They and others have suggested that increased activity above motor regions may reflect a central effort to counteract cortical or peripheral loss of force production capability during repetitive contractions [15,23,24,26,38].

Others have observed decreases in cortical activity during or after fatiguing muscle contractions. In an investigation of handgrip exercise at 40% MVC, fMRI activity decreased across the primary sensorimotor cortex, supplementary motor area, cerebellum, and visual cortex [20]. Yang *et al.* [19] observed sensorimotor decreases (EEG) during the sustained phase of a handgrip contraction at 20% MVC [19]. Decreased activity was not seen at higher loads, however: sensorimotor activity (EEG) increased with fatigue in a grasping task at 70% MVC [23]. Freude and Ullsperger [25] examined motor activity directly before contractions at 80%, 50%, and 20% of MVC and noted increasing activity with fatigue at 80% but not with the non-fatiguing 50% protocol. Thus high-volume movements appear to increase cortical activity when loads are relatively high, although one investigation at 100% MVC had differing results: an initial increase in several regions (fMRI) was eventually followed by decreases in motor, prefrontal, and cingulate activity that coincided with decreased force [38]. This was suggested to reflect “an early adjustment to strengthen the descending command for force-loss compensation and subsequent inhibition by sensory feedback as fatigue became more severe”. 

Sensory feedback may explain why loading and volume appear to influence the direction of cortical activity during fatiguing conditions. Others have observed decreases in motor cortical activity with training [28] and when comparing trained and untrained subjects [31]; based upon these investigations, we can speculate that relatively lower levels of familiarity with fatiguing high load schemes may result in increased cortical activity. These increases could reflect the attempt to compensate for decreasing force and increasing information from the periphery with fatigue. Sensory activity mirrored motor activity with regards to our experimental protocols: the highest levels accompanied protocols where fatigue (and ostensibly feedback from the periphery) was highest. While cortical activity increases under these circumstances, it is interesting to note that this increased activity did not result in increased muscular force. This may be due to intrinsic changes in the skeletal muscle (e.g., damage, metabolic, adrenergic, or acidic environment). 

## 4. Experimental Section

### 4.1. Design

Following a familiarization and first visit, a balanced, randomized, within-group design was used. Four exercise visits, including a control protocol (CTRL), were spaced one week apart and randomized with respect to sequence. Electroencephalogram (EEG) recordings were obtained throughout each protocol visit, and data recorded during each complete repetition was used to produce quantitative data on regional cortical activity increases in the cortex. Blood, perceptual measures, and performance data were also collected to better characterize the stimuli accompanying each protocol.

The *squat* exercise was chosen for its prominence in daily living and training as well as its adaptability to differing biomechanical properties. From a standing posture, the pattern of bending at the hip and knee for the purpose of lowering the body to a reference position (such as sitting in a chair, rising from a resting position, lifting and carrying) is typically throughout the day. In addition to the use of squat variations in everyday activities, the resistance exercise program of a highly-trained individual is likely to incorporate thousands of loaded squat repetitions in a given year. The squat is easily adapted to emphasize various biomechanical properties (e.g., force, power, or volume), which makes it ideal as an experimental movement. Equally important, when performed repetitively with maximal exertion, the squat incorporates large amounts of muscle activation and creates substantial fatigue [35,39,40,41]. In addition to large involuntary losses in force production capability, fatigue is characterized by physiological responses that include large increases in circulating corticosteroids, catecholamines, androgens, markers of metabolic activity and tissue disruption, heart rate, and perceptual stress [42,43,44,45,46,47]. This investigation thus utilized the squat exercise movement as a highly-trained, dynamic movement. 

### 4.2. Subjects

**Figure 4:**
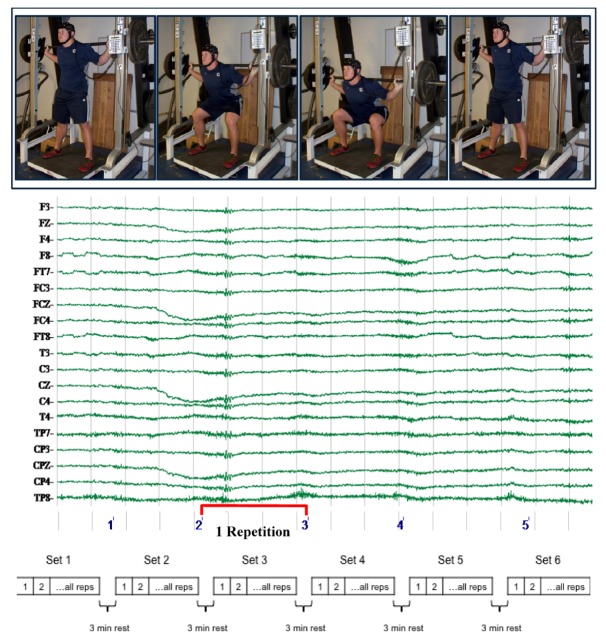
Experimental Movement and Design Process. Subjects completed 6 repetitions of the squat movement at the prescribed load for each set. All protocols included 6 sets with three minutes of rest between.

Since subjects were required to perform resistance exercise at loads approaching maximal voluntary dynamic force with minimal learning effects, only those who were highly-trained in the squat exercise were included. As a result, the training state of the subjects was rare by comparison to general or recreationally trained populations. Potential subjects were screened to ensure continuous participation in *intense* resistance squatting exercise; all subjects used of loads in excess of 80% of 1RM at least once a week in their training. In addition, they practiced resistance exercise for a minimum of four continuous years (our population averaged 6 ± 1 years). Subjects demonstrated the ability to proficiently perform a standardized squat with an added load of at least two times their body mass on a Smith machine. The smith machine was selected because it allows subjects to perform a squat while the bar is fixed on a vertical track. The track permits movement in the sagittal plane while lessoning movement of the bar in the transverse plane, allowing investigators to directly measure velocity, power, and displacement with a linear transducer. The average squat load for their one-repetition maximum (1RM) was later determined to be 174 ± 26 kg at their first visit (the Smith machine apparatus is pictured in Figure 4). Potential subjects completed a comprehensive medical history that prohibited the use of benzodiazepines, anti-epileptic and seizure medications, or anabolic steroids; a physician cleared participating subjects for an absence of disease and fitness for vigorous exercise.

At the conclusion of the screening process, healthy, highly-trained, non-smoking men (*n* = 7; height: 175.01 ± 7.07 cm; body mass: 85.76 ± 9.86 kg; age 22 ± 3 years) volunteered to participate in the investigation. All enrolled subjects attended a one-hour verbal explanation of the study protocol, had the associated inconveniences and risks explained to them, had any questions answered, and voluntarily signed the consent document. The local Institutional Review Board approved all procedures for use of human subjects.

To account for individual and collective variation, 24 h prior to all visits (including the familiarization and first visit), subjects replicated diet, water intake, caffeine use, and a breakfast while abstaining from alcohol. No statistical differences were noted between subjects or within visits for hydration (urine color < 5, USG < 1.025), life stress (Holmes-Rhea = 0), sleep quality, or hours of sleep. Each subject maintained a specified testing time in the morning. Subjects abstained from exercise for 48 h (72 h for lower body exercise) prior to each protocol visit. 

### 4.3. EEG Data Acquisition

Cortical activity was measured using an Ag-AgCl electrode-containing elastic nylon cap in conjunction with a 40-channel monopolar digital Amplifier (NuAmps, Neuroscan, Inc., El Paso, TX, USA). Cap electrode configuration was set to an expanded version of the international 10-20-10 electrode placement system [48]. A linked-ears reference scheme was used with a separate scalp electrode serving as a ground. To monitor and account for electrical artifacts stemming from eye movements, blinks, and facial muscle contractions, electrodes were placed two centimeters above and below the left eye as well as horizontal to each pupil. Scalp preparation (hair was not shaved) included abrasive cleansing without the use of conditioner the morning of data collection, manual abrasion with a hard-bristle comb by research staff, and gentle manual abrasion with a blunt-nose syringe prior to the injection of conducting gel into each electrode. Abrasive prep-gel and alcohol was also used as appropriate.

Cortical activity was recorded continuously using Neuroscan’s Scan 4.4 Software. A 32-bit analog-to-digital converter was used to digitize signals from each channel at a sampling rate of 1000 Hz. High and low pass filters were set at DC and 100 Hz, respectively. Impedance was monitored and kept *at or below 5 kΩ* throughout all experimental protocols. All recordings took place with eyes-open [49]. Visual event markers were used to signify the beginning and completion of each repetition throughout each protocol. A quiet, cool, and low noise environment was used throughout experimental protocols to minimize environmental stimuli, perspiration, and background electrical noise.

### 4.4. Familiarization and Simulation Visits

During the familiarization visit, all study protocols and procedures were explained in detail. Protocols and procedures were practiced at various loads and the control day procedure reviewed until staff determined that subjects were able to correctly perform all tasks. The purpose of the first visit was to simulate experimental procedures and to obtain squat 1RMs (which were used to assign resistance loads in experimental protocols). To obtain 1RMs, single repetitions of the squat were performed at increasingly heavier loads as previously described [35]. The test was stopped when the subject could no longer increase the load on the bar while squatting to a reference parallel position.

### 4.5. Experimental Visits

The resistance load, set, and repetition schemes for the PWR, FOR, and VOL protocols are reported in Table 3 (see Figure 4 for a visual depiction of sets and repetitions). Subjects completed every aspect of the experimental protocols during the CTRL protocol with the exception of movement. Subjects un-racked the unloaded (147 N) resistance bar as they ordinarily would for each set and stood silently in the exercise apparatus for twenty seconds before re-racking the bar. The mean force for CTRL (975.38 ± 100.32 N) represents the mean body mass with the added weight of the bar. Three minutes of rest between sets were used for all protocols.

**Table 3:** Physical Characteristics of Experimental Protocols. Rest period was three minutes between sets. Reps = repetitions of the squat movement; F = force; Vel = velocity; M = mass; A = acceleration due to gravity; D = displacement. The mean one-repetition maximum (1RM) was 174 ± 26 kg.

Protocol	Sets	Reps per Set	Load	Objective
**PWR**	6	3	30% 1RM	Generate maximal repeated mechanical power (F × Vel)
**FOR**	6	3	95% 1RM	Generate maximal repeated dynamic force (M × A)
**VOL**	6	10	80% 1RM	Generate maximal number of high-load repetitions (F × D)
**CTRL**	6	N/A	15 lbs (bar)	Stationary stand for 20 s

**Figure 5:**
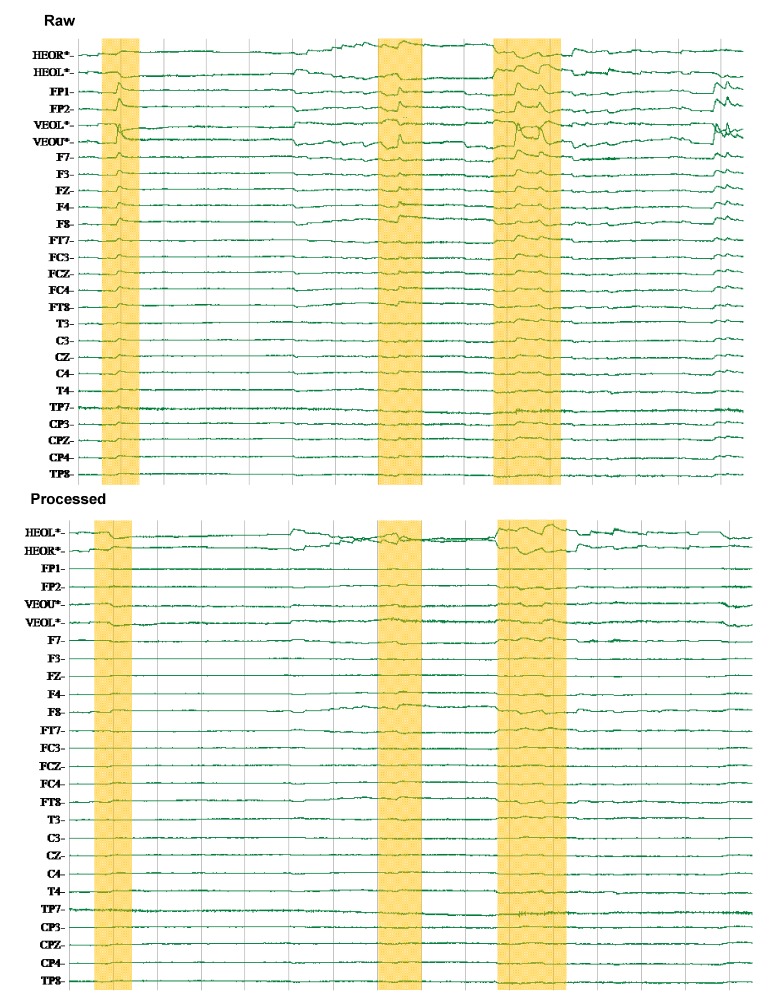
Artifact Removal: Before and After Processing. Signals were processed to remove potential artifacts from facial and eye muscle contractions using spatial and frequency-based filters.

### 4.6. Biomechanical Properties

Data were collected with an integrated force (Fitness Technology 400 series performance force plate, Australia) and linear transducer (Celesco, Chatsworth, CA, USA) apparatus. All data were reported using Ballistic Measurement System software (Software Version 2009.0.0). Equipment was internally and externally calibrated prior to use and data were sampled at 200 Hz. Parallel squat depth was set to a displacement of zero and confirmed as appropriate.

The PWR protocol incorporated maximal loaded mechanical lower-body power, which is developed at 30% 1RM in the ballistic Smith squat exercise [37,50]. The mean resistance load used for the PWR protocol was 52.27 ± 8.09 kg and the power output was significantly greater than all other protocols. The FOR protocol incorporated near maximal dynamic loading (95% 1RM); the mean resistance load used was 164.94 ± 24.23 kg with significantly greater force than all other protocols. A high VOL protocol maximized total work and fatigue by incorporating heavy loading over the greatest number of repetitions (80% 1RM for 6 sets of 10 repetitions). VOL used 138.31 ± 20.43 kg and had greater total volume and work than all other protocols. Mean force in the VOL protocol was similar to mean force in the PWR protocol with the difference in power production attributable to the higher velocity of PWR. Likewise, mean velocity in the FOR protocol was similar to mean velocity in the VOL protocol with the difference in power attributable to the higher loads of FOR.

### 4.7. Measures of Exertion and Physiological Impact

The CRQ-10 scale [51,52] was used to measure perceived exertion (from 0 to 10 or higher if appropriate). Blood measures were collected to assess the physiological impact of experimental protocols. Lactate values were measured using a STAT 2300 (Yellow Springs, Inc., Yellow Springs, OH, USA) and used as a global indicator of metabolic stress [53]. 

### 4.8. EEG Signal Processing and Analysis

Raw, continuous EEG signals were processed to produce final data (Edit, Compumedics Neuroscan, El Paso, TX, USA). All continuous data files were visually checked for gross artifacts; channels possessing such artifacts were eliminated (Figure 5). Artifacts created by eye movement and facial muscle contractions were removed with a spatial filter, which used a spatially conscious covariance matrix to remove artifacts without negatively attenuating co-varying brain-generated signals (particularly in pre-frontal and frontal regions). 

Continuous data were epoched from the beginning of the eccentric component until the completion of the concentric component of every repetition. An infinite impulse response filter (set as a 50 Hz low pass filter with a roll-off of 12 dB/oct) was applied to all data. The mean rectified amplitude of electrode activity was grouped by region (Premotor—FC3, FCZ, FC4; Primary Motor—C3, CZ, C4; Sensory—CP3, CPZ, CP4; Parietal—P3, PZ, P4). Previous work has depicted CZ as the supplementary motor area and C3 as the sensorimotor cortex [9] for clarity, we have described both as primary motor based on general consensus in the literature [54]. A three-dimensional integral mapping function was used to spatially depict the most characteristic cortical activity during a repetition in each protocol. 

Few investigations have obtained EEG during dynamic maximal exertion movements so there was a concern over whether noise might be present in our signals. The native signal-to-noise (SNR) function of the Neuroscan Edit program was used to compute SNRs for each protocol and overall. For the first repetition of each set, the noise from −1000 to −500 ms prior to each repetition (during which time the subjects were standing in the rack with the full load on their back) was compared to the signal of the entire repetition itself for all electrodes in our analysis. The average signal-to-noise ratio across all protocols was 8:1.

### 4.9. Statistical Analyses

Quantitative values are presented as means and standard deviations (SD). Significance in this study was set at *P* ≤ 0.05. A log10 transformation was applied to data and assumptions for linear statistics confirmed or corrections applied as applicable. A three-way within-group (protocol × set × region) analysis of variance (ANOVA) with repeated measures (main effects suppressed) was used to detect significant mean differences. Planned pairwise comparisons of interest with Fisher’s LSD were used to discern the nature and significance of differences between variables. 

## 5. Conclusions

We found that increases in functional cortical regions during the fatiguing VOL protocol greatly overwhelmed increases seen in PWR and FOR. The sensory demands of the PWR protocol were reflected in high motor and sensory activity during the first set. However, activity in these regions did not increase as it did in the VOL protocol; if anything, the activity in PWR decreased over time. While the biomechanical properties of movement were differentially represented in regional cortical activity, amplitude, and patterns, cortical activity appeared to reflect fatigue. Since fatigue would by definition appear to reflect the most acute peripheral feedback, it was not surprising to observe the greatest levels of and increases in sensory cortical activity in the protocols where fatigue was greatest. Fatigue may be an important stimulus, indicator, and goal for exercise prescriptions. These data may provide additional insight into the relationship between muscle performance and motor/sensory activity in cortical regions of the brain.
